# Carbonate Micromotors for Treatment of Construction Effluents

**DOI:** 10.3390/nano10071408

**Published:** 2020-07-19

**Authors:** Purnesh Chattopadhyay, Priyanka Sharan, Andrej Berndt, Juliane Simmchen

**Affiliations:** 1 Chair of Physical Chemistry, TU Dresden, 01062 Dresden, Germany; purnesh.chattopadhyay@mailbox.tu-dresden.de (P.C.); priyanka.sharan@tu-dresden.de (P.S.); 2 Implenia Schweiz AG, CH-8304 Wallisellen, Switzerland; andrej.berndt@implenia.ch

**Keywords:** pH neutralization, spray coating, active matter, carbonate micromotors

## Abstract

Concrete in construction has recently gained media coverage for its negative CO_2_ footprint, but this is not the only problem associated with its use. Due to its chemical composition, freshly poured concrete changes the pH of water coming in contact with the surface to very alkaline values, requiring neutralization treatment before disposal. Conventional methods include the use of mineral acid or CO_2_ pumps, causing high costs to building companies. In this paper, we present a micromotor based remediation strategy, which consists of carbonate particles half-coated with citric acid. To achieve this half coverage spray coating is used for the first time to design Janus structures. The motors propel diffusiophoretically due to a self-generated gradient formed as the acid coverage dissolves. The locally lower pH contributes to the dissolution of the carbonate body. These motors have been employed to study neutralization of diluted concrete wash water (CWW) at microscopic scale and we achieve visualization of the pH changes occurring in the vicinity of motors using anthocyanine as pH indicator dye. The effect of citric acid-carbonates hybrid on neutralization of real CWW on macroscopic scale has also been studied. In addition, all employed chemicals are cheap, non-toxic and do not leave any solid residues behind.

## 1. Introduction

Micromotors and self-propelling particles have been suggested for various environmental remediation purposes, especially in wastewater treatment [1,2,3].

Different functionalities and inherent capacities of materials have been used in this regard; mainly adsorption and disposal [4], mineralization through catalytic processes [5] and highly specific interactions have been employed. Adsorption generally requires surface interactions, for which a high surface area is beneficial. Therefore, carbon based materials [6], especially graphene [7,8] have been used frequently but also other porous materials are employed [9]. Surface modification can enable more specific interactions such as the binding of heavy metals through chelating agents [10]. Different removal strategies could also be achieved through more specific interplays, as has been demonstrated for hydrophobic interactions enabling the collection of oil droplets [11,12].

The complex combination of chemical and hydrophobic interactions [13] enabled Au@Ni@TiO_2_ micromotors to efficiently remove microplastics [14]. Even though adsorption is a useful and cost-friendly process, it merely involves the accumulation of contaminants, not their degradation. Catalytic decomposition of pollutants to harmless products (mineralization) can be achieved using advanced oxidation processes, such as the iron based Fenton and Fenton-like processes in peroxide, commonly used in wastewater treatment [15]. Coupling these processes with active colloids leads to efficient dye degradation [16,17,18,19], but also other dangerous substances such as radioactive waste, warfare agents or nitro-aromatic compounds could be mineralized through radical formation in the catalytic processes [20,21].

However, the fabrication of most micromotors involves multi-step clean room production, often expensive noble metals and complicated devices [16]. Several endeavours like electrodeposition and template free approaches have been presented but often still rely on precious metals [17].

Many of the environmental problems happen globally and suggested solutions should be scalable, cheap and easy to manufacture.

**Figure 1:**
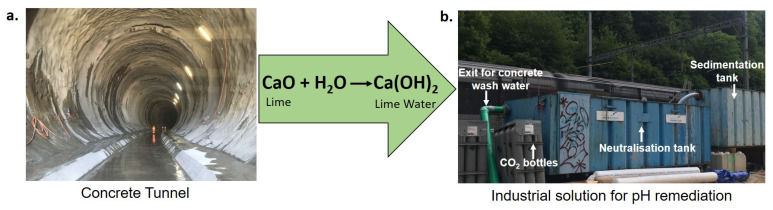
(**a**). Lime which is a key component of concrete, reacts readily with water to produce slaked lime Ca(OH)_2_. It solubilizes sparingly in water to produce highly alkaline solutions. (**b**). Industrial CO_2_ based pH reduction: the CWW first enters a sedimentation tank in which most of the solids, sludge and dirt settle down and the water flows over these surfaces in into pump pits. In Europe, regulations allow maximum turbididty, which is achieved by sedimentation. In a neutralization tank the pH of CWW is reduced by purging CO_2_ from adjacent CO_2_ bottles. Finally, the CWW with neutralized pH exits from the tank.

Concrete is a mixture of cement, aggregate (sand and gravel) and water [22]. A global concern of using concrete in construction is that cement production contributes to 7% of the worlds CO_2_ emission [23,24,25,26]. Due to its chemical composition (see Figure 1), freshly produced concrete also causes pH increase in water which lasts for years after fabrication. Concrete wash water (CWW) mainly comes from the water that was in contact with newly made buildings and from water used for cleaning concrete trucks. Due to the presence of high amounts of dissolved CaO and other hydroxides, the pH ranges from 11 to 12, making it toxic for fish and other aquatic life [27]. Consequently, many regulations have been imposed on industries and construction companies to handle CWW properly before disposal [28] and neutralization is extremely important. Mineral acids, citric acid and carbon dioxide are the most common reagents used for this purpose [29], each coming with advantages and disadvantages. For example, mineral acids are dangerous to handle and precise pH control is difficult. Carbon dioxide suffers the disadvantage that the initial setup and maintenance costs are very high as compared to normal acid injection [29].

In western countries, these remediation processes are usually taken care of (see Figure 1) and processed, pH neutral water is disposed into a discharge system (streams, rivers or sewers) or sometimes via drainage shafts directly enters the ground (see Section S1 and cost overview in SI). However, these processes are costly, intense in maintenance and often cause problems, so companies still look for cheaper and more easily implementable solutions. In developing countries often the pH is reduced only by dilution with water. Dilution clearly is not a solution because pH scales logarithmically and thus 10-fold dilution would be required for one unit reduction in pH.

Here, we present a novel micromotor which is based on carbonate particles [30], which are half coated with citric acid via spray coating. This novel type of asymmetrization allows to create a residue-free gradient for propulsion and also for pH remediation of alkaline wash water from concrete. All materials are cheap and eco-friendly. Compared to other methods such as CO_2_-based neutralization, our approach does not require any external power supply, nor expensive setups for operation. In contrast to using mineral acids, the combination of citric acid and carbonates is safer to use and helps to neutralize the wash water rather than just lowering the pH. The carbonate based micromotors are efficient in controlling the overdosage of acid into the solution, and completely decompose into harmless metal citrate and CO_2_.

## 2. Experimental Section

### 2.1. Materials and Reagents

Calcium chloride dihydrate (99%), anhydrous sodium carbonate (99.5%), Poly(sodium 4-styrenesulphonate) (M.Wt. 1000 KDa) were purchased from Sigma-Aldrich (Germany). Manganese sulfate monohydrate was purchased from Bernd Kraft. Ammonium hydrogencarbonate (99.5%) and citric acid (99%) were purchased from Grüssing GmbH; 250 nm SiO_2_ particles were obtained from G. Kisker GbR. Fresh red cabbage was bought from local supermarket. Sand (with small granules and pebbles) and cement were obtained from a construction site. Ultrapure water was used which was obtained from in-house millipore water purification system. All chemicals were of analytical grade and used without any further purification.

### 2.2. Methods

Zeta potential and size measurements were performed using Malvern Zetasizer Nano ZSP, partially in autotitration mode using the attached Multi Purpose Titrator. The titration was carried out from basic to acidic pH using either HCl or citric acid as titrant and zeta potential or size values were noted at every 0.5 pH unit interval. Before each measurement the sample was equilibrated for 120 s at 25 ${}^{\circ }$C.

Powder X-ray diffraction (XRD) was performed using Bruker 2D phaser in 2θ range of 10–100${}^{\circ }$. Cu source operating at 30 kV and 10 mA was used for the measurement. The particles were dispersed in ethanol and were subsequently drop casted on a Silicon wafer. Data analysis was performed using WinXPow software with Inorganic crystal structure references.

Scanning electron microscopy (SEM) images were obtained using Zeiss DSM 982 GEMINI electron microscope using 4 kV electron beam. For individual particles, they were dispersed in ethanol and drop casted on Aluminium sample holders. Images of monolayers were taken directly after depositing them on Silicon wafers.

UV-Visible spectroscopy (UV-Vis) of samples dispersed in water was performed on Cary 50 Scan UV-Visible spectrophotometer in 400–700 nm wavelength range using a 1 cm path length quartz cuvette.

Fourier transform infrared spectroscopy (FTIR) was performed on Thermo Scientific - Nicolet 8700 with attached Thermo Scientific Smart iTR in Attenuated Total Reflectance (ATR) mode. Powder samples were used for the measurement.

Motion studies were done using Zeiss camera attached to an inverted microscope (Carl Zeiss Microscopy GmbH Germany). Zen 2.3 software was used for recording the videos. Basler ace colored camera and Pylon viewer software were used for microscopic scale neutralization experiments. Video recording was done at 40 frames per second rate at different magnifications. Analysis of the videos were done using ImageJ software and Trackmate plugin.

### 2.3. Preparation of CaCO_3_ Microspheres

CaCO_3_ was prepared by a coprecipitation method using CaCl_2_ and Na_2_CO_3_ by following the literature with some modifications [31]. Briefly, 0.1 g of Poly(sodium 4-styrenesulphonate)(PSSS) was added to 100 mL of 16 mM Na_2_CO_3_ solution and subjected to stirring until complete dissolution. The pH of this solution was adjusted between 8 to 12 using 1 M NaOH solution. Thereafter, 3.2 mL of 1 M CaCl_2_·2H_2_O solution was rapidly injected into the mixture under ultrasonication. The final mixture was then stirred for 1 min and kept overnight for settling and further growth. The particles were washed with water three times and were separated by centrifugation. The sample was dried at 50 ${}^{\circ }$C overnight.

### 2.4. Preparation of MnCO_3_ Microcubes

Controlled synthesis of cubic MnCO_3_ was done by following the literature [32] with some modifications. First, a 20 mL nano-seed solution was prepared using 0.1 mg of MnSO_4_·H_2_O and 4 mg of NH_4_HCO_3_ by homogeneous mixing. Then, 0.2 mL of this solution was rapidly injected into 100 mL of 6 mM MnSO_4_·H_2_O (with 0.5 *v*/*v*% isopropanol). To this above mixture 100 mL of 0.06 M NH_4_HCO_3_ with 0.5 *v*/*v*% isopropanol was added under ultrasonication. The bath was maintained for 15 more minutes, and finally rested for 30 min to form particles. The particles were washed thoroughly with water 4–5 times and then dried at 60 ${}^{\circ }$C overnight.

### 2.5. Preparation of Janus Structure by Acid Layer Coating

An acid layer was coated on the carbonate particles to create an asymmetry for propulsion and also to change the pH. Typically a uniform packed monolayer of the carbonate particles was prepared using Langmuir-Blodgett thin film deposition technique on a glass substrate. The monolayer was then spray coated with citric acid in ethanol with a flow rate of 0.6 mL/h and the distance between nozzle and bed was kept 45 mm. For CaCO_3_ monolayers, 4 layers of 0.0185 M citric acid and for MnCO_3_ monolayers, 2 layers of 0.185 M citric acid (in accordance with the solubility of citric acid in ethanol) were used. Finally, citric acid coated CaCO_3_ micromotors (CA@CaCO_3_) and citric acid coated MnCO_3_ micromotors (CA@MnCO_3_) were obtained. Very fast dissolution of CA@CaCO_3_ motors compelled to use a 10 times dilute concentration of citric acid for CaCO_3_ monolayers.

### 2.6. Preparation of Concrete Wash Water

Small structures of concrete were made using cement, granular sand and water in a weight ratio of 2:1:3 and dried. These structures were kept in deionized water overnight. The supernatant obtained (later referred as CWW) had an initial pH of 11.2 which was used for experiments either directly or after required pH adjustments by dilution.

### 2.7. Motion Studies of (CA@CaCO_3_) and (CA@MnCO_3_) Micromotors

A small amount of these micromotors were scratched on a plasma cleaned glass slide and subsequently either deionized water or diluted CWW (pH 9) or a NaOH solution (pH 9) was added. The motion of the micromotors was recorded using attached Zeiss camera to an optical microscope at 40 frames per second. The speed of the micromotors were shown in a box plot, giving the minimum, maximum and the interquartile range marking the 5th, 25th, 75th and 95th percentile of the micromotor speed. The middle line and small box represents the median and mean respectively. Motion statistics of over 50 micromotors per case were taken and plotted.

### 2.8. Preparation of Anthocyanin Dye

Typically 100 g of red cabbage was macerated without any solvent in a mortar-pestle. Resulting extract was centrifuged and used in experiments. Fresh samples were prepared before each use.

### 2.9. Neutralization Experiments of Concrete Wash Water on Microscopic and Macroscopic Scale

A 20 μL droplet of diluted CWW (pH 9) with red cabbage indicator (5 *v*/*v*%) was imaged using an optical microscope. The changes after subsequent addition of CA@CaCO_3_ or CA@MnCO_3_ micromotors were observed frame by frame. For macroscopic scale neutralization experiments a series of autotitrations were done. Briefly, 12 mL of filtered CWW (pH 11.2) was taken with 0.085 *w*/*v*% of either CaCO_3_ or MnCO_3_ particles. Additionally, 250 nm SiO_2_ particles were added as tracers to the above mixture for determination of zeta potentials. This mixture was titrated from basic to acidic pH using 0.2 M citric acid solution while simultaneously measuring zeta potential using the attached Malvern Zetasizer Nano ZSP. Freshly prepared citric acid solution was used for every titration. For comparison, one more set of titration was carried out without any carbonate particles. Each titration was repeated at least three times to ensure reproducibility.

## 3. Results and Discussion

**Figure 2:**
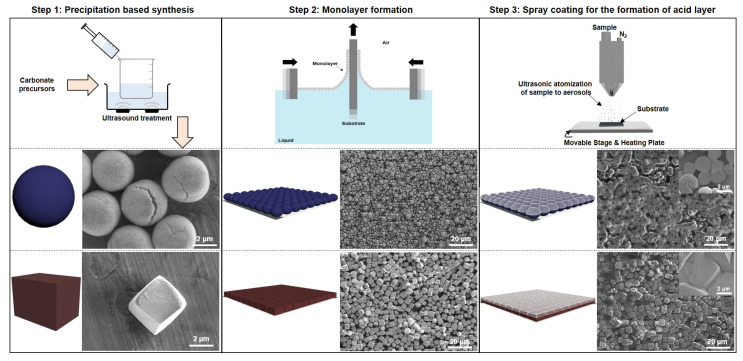
Steps involved in the fabrication of micromotors with corresponding illustrative and SEM images. Step 1: Co-precipitation employed for the synthesis of spherical CaCO_3_ and cubic MnCO_3_ particles. Step 2: Monolayers of these particles were formed using Langmuir-Blodgett method. Step 3: Citric acid was spray coated on the monolayers to form CA@CaCO_3_ and CA@MnCO_3_ micromotors. The inset SEM images of citric acid coated monolayers were obtained at higher magnification.

To produce the bodies of carbonate micromotors, spherical shaped CaCO_3_ and cubic MnCO_3_ are synthesized via a simple co-precipitation method. Subsequently, these particles are deposited into a monolayer using a Langmuir-Blodgett trough (see Figure 2). To confer asymmetry to the particles we developed a novel, clean room free, easily scalable strategy: the particle monolayers are spray-coated with an ethanolic citric acid (CA) solution, which then quickly evaporates upon deposition and crystallizes, guaranteeing a homogeneous deposition of citric acid on the carbonate particles. This avoids premature pH induced dissolution of the carbonate materials. This method creates an asymmetry within the particles, necessary for self propulsion.

In previous reports Co@CaCO_3_ micromotors were moving in an acidic gradient created by HeLa cells [33]. Here, neither additional metal (noble) layers, nor permanently remaining materials in the micromotor body are required, only citric acid as widely accessible, inexpensive chemical is used for asymmetrization. This process also makes the micromotors independent of external gradients. Schematic images of the steps involved in the fabrication are shown in Figure 2 and in the supplementary information (Section S2).

The motion of CA@CaCO_3_ and CA@MnCO_3_ micromotors were studied and compared in different media. Preliminary, motion was observed in water. Upon contact with water, micromotors start to move due to acid dissolution and reaction of carbonate and citric acid as shown in Equation (1).

(1) $$2C_{3}H_{5}O{\left(COOH\right)}_{3}+3MCO_{3}\overset{H_{2}O}{\to }M_{3}{\left(C_{6}H_{5}O_{7}\right)}_{2}+3CO_{2}+3H^{+}+3OH^{-}$$

where M: Ca^2^+ or Mn^2^+.

**Figure 3:**
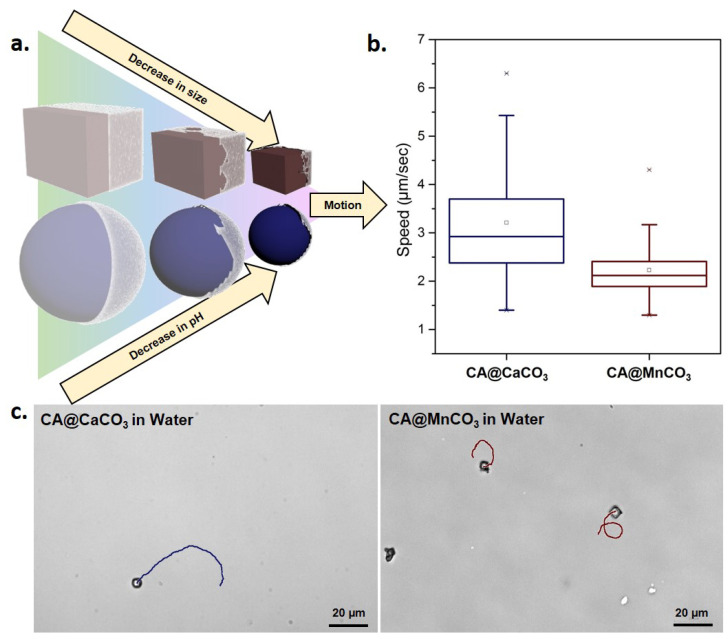
(**a**). Scheme illustrating the concept of propulsion of CA@MnCO_3_ and CA@CaCO_3_ micromotors: The transparent layer on the particles depict citric acid coating. The color gradient from green to pink indicates the pH change that happens due to citric acid dissolution. The reaction between carbonate and citric acid causes shrinking of the micromotors and subsequently, a forward motion. (**b**). Speed of CA@CaCO_3_ and CA@MnCO_3_ micromotors in water. (**c**). Tracks of CA@CaCO_3_ and CA@MnCO_3_ micromotors in water.

This self-degrading reaction generates a gradient around the motors forcing them to propel diffusiophoretically, other authors have also coined the term chemokinesis for propulsion due to dissolution [34,35]. The motion allows effective diffusion of protons, leading to complete and efficient neutralization. As shown in Figure 3b, the speed of spherical CA@CaCO_3_ is comparatively higher than cubic CA@MnCO_3_. A possible explanation originates either from the spherical shape which is hydrodynamically more favorable or alternatively, the higher dissolution rate of CA@CaCO_3_ compared to CA@MnCO_3_ (discussed more in SI (Section S4)). From the tracks of these micromotors we deduce that the motion is of random direction and they move with an average speed of 2.5 to 3.5 μm/s which is slightly slower than catalytic Pt@SiO_2_ micromotors of comparable size (Figure 3c) (see supporting Video S1) [36].

The reaction of citric acid with carbonates causes a decrease in size of these micromotors and additionally creates a pH gradient around them which is schematically shown in Figure 3a and further discussed in SI (Section S4). The gradient is color-coded in agreement with the anthocyanin indicator, as an example of a cheap, easily available and environmentally friendly dye with a broad range of colors: green being most basic and red corresponds to acidic values.

**Figure 4:**
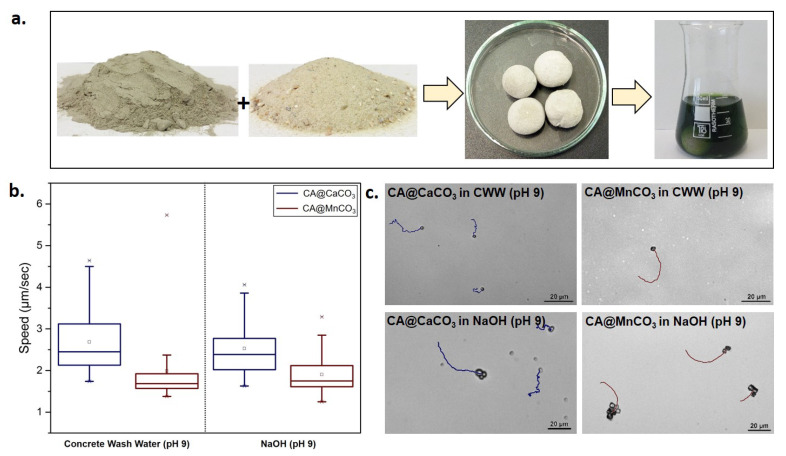
(**a**). Preparation of concrete wash water (CWW). Concrete structures made from a mixture of cement, granular sand and water were immersed in DI water containing anthocyanin dye. The green color indicates high alkalinity of CWW. (**b**). Speed of CA@CaCO_3_ and CA@MnCO_3_ micromotors in CWW (pH 9) and NaOH (pH 9). (**c**). Tracks of CA@CaCO_3_ and CA@MnCO_3_ micromotors in CWW (pH 9) and NaOH (pH 9).

The motion of these micromotors in a diluted CWW sample (prepared as shown in Figure 4a) of pH 9 and in a comparable NaOH solution (see supporting Videos S2 and S3) shows that CA@CaCO_3_ and CA@MnCO_3_ micromotors move in this high pH medium at a lower speed in comparison to pure water (at almost neutral pH) as shown in Figure 4b with corresponding tracks in Figure 4c. We associate this behaviour to the high ionic strength of concrete and NaOH solution, conductivity values of different mediums are given in SI (Table S2). Motion studies in CWW (pH 11.2) could not be conducted due to its high ionic strength limiting the propulsion of these micromotors [37,38].

**Figure 5:**
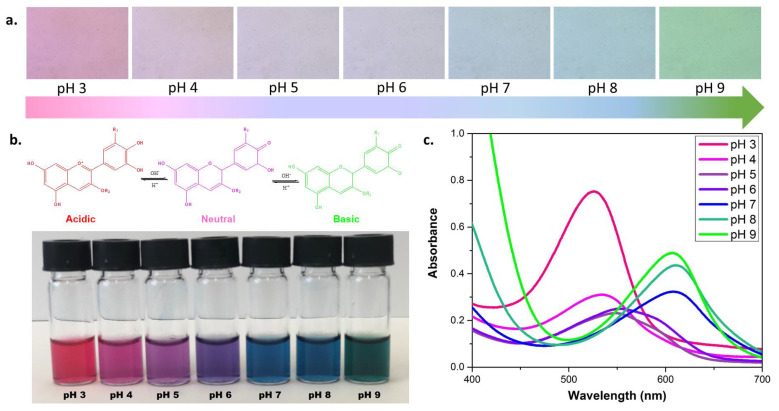
(**a**). Microscopic images of anthocyanin dye in different pH buffer solutions. (**b**). pH dependent structures of anthocyanin dye molecule with corresponding bulk images. (**c**). UV-Visible spectrum of red cabbage indicator in different pH solutions.

The extracted anthocyanin dye from red cabbage appears in different structural conformations, depending on the pH (Figure 5b). These structural changes lead to different degrees of confinement of electrons within the structure, resulting in different colors [39]. The color changes of the extracted indicator at different pH buffer solutions from 3-9 are presented in both, bulk and microscopic dimensions. In Figure 5a the relative microscope images of indicator in different pH buffers is shown. For the bulk images, 200 μL of freshly prepared indicator were added to 1 mL of buffer of different pH. Both, bulk and microscope images correlate with each other. To confirm the visual changes obtained in the microscope, UV-Visible spectra of red cabbage indicator for diluted samples are displayed in Figure 5c. The relative absorption intensity and maximum absorption wavelength of the dye shift with different pH values.

**Figure 6:**
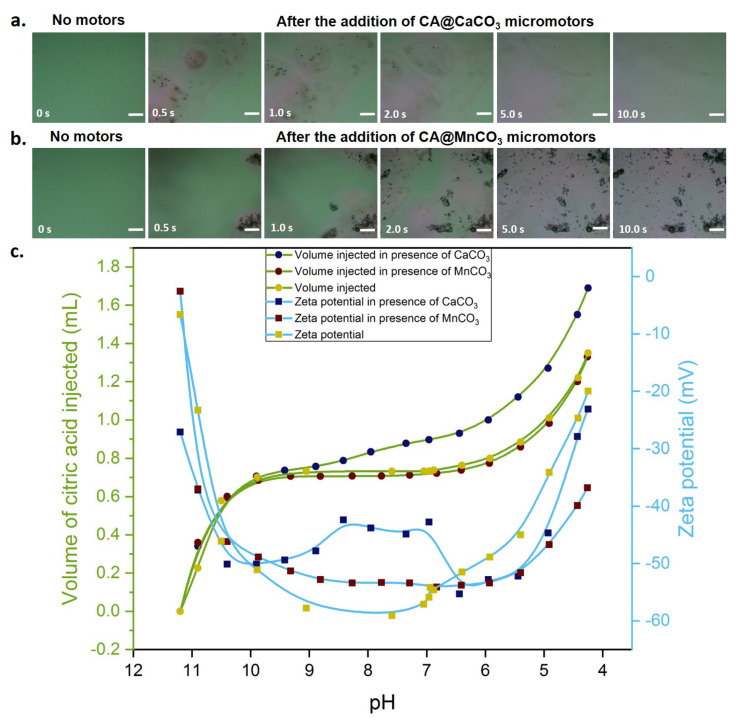
(**a**,**b**). Time lapse optical images of diluted CWW (pH 9) with red cabbage indicator. The image at 0 s was captured with no motors and after that either CA@CaCO_3_ or Ca@MnCO_3_ micromotors were added and subsequently images were captured. (**c**). Titration of CWW (pH 11.2) with citric acid solution in presence or absence of either CaCO_3_ or MnCO_3_ particles and corresponding zeta potential values. Scale bar is 100 μm.

The effect of hybrid citric acid-carbonate particles in neutralization of CWW was studied in both, microscopic and macroscopic scale. The optical images in Figure 6a,b show the neutralization with time after the addition of the CA@CaCO_3_ or CA@MnCO_3_ micromotors respectively. Without micromotors, the droplet remains green in color (showing high alkalinity (pH 9)). After addition, the pH in vicinity of motors gets reduced rapidly which can be observed from the progressive color changes in Figure 6a,b (see also supporting Video S4). With the increase in time, the motors move and get dissolved, thus enhancing the diffusion and achieving a complete reduction of pH.

Technically, it is impossible to disperse the micromotors in water prior to use since their inherent structure would immediately lead to motion and therefore dissolution. To guarantee an analytically reliable measurement, weighing of small quantities of micromotors required for macroscopic neutralization experiments had to be avoided. Therefore, to evaluate the required quantities on the macroscale and enable us to calculate the associated cost, we performed a titration of CWW (pH 11.2). This titration was done with citric acid either in presence or absence of CaCO_3_ or MnCO_3_ particles to demonstrate the effect of carbonate particles on the neutralization of CWW. Tracer silica particles are added to determine zeta potentials.

One has to take into account, that the measured zeta potentials at high pH values are strongly influenced by the carbonate particles. In the zeta potential curve for CaCO_3_ particles the behaviour in between pH 9 and pH 6 reflects the dissolution of the CaCO_3_ particles (See Figure S5c). The trend of zeta potential of SiO_2_ particles is consistent with the literature [40]. As can be seen from Figure 6c, in all three cases the pH of CWW decreases with injection of citric acid solution. However, comparing the slopes of the titration curves indicates that in presence of CaCO_3_ particles, the required volume to achieve a certain pH change is higher than in both other cases (presence of MnCO_3_ particles and absence of carbonates). This effect can be associated to CaCO_3_-citric acid reaction that produces H_2_CO_3_ and has a buffering effect and thereby avoids ’over-acidification’. Figure S5a,c in SI confirm this effect. CaCO_3_ particles dissolve significantly faster than MnCO_3_ and hence this buffering effect is more pronounced for CaCO_3_. Thus, over dosage of citric acid in neutralization can be controlled by using a hybrid of CaCO_3_-citric acid.

## 4. Conclusions

An increasing population today still imposes a general problem to hydrological ecosystems. Along with numerous factors such as unprocessed waste water from households, alkaline wastewater from construction sites can turn fresh water supply into a serious problem, imposing a serious burden to receiving waters and aquatic species living therein. To remediate these effects, in most countries the construction industry is officially obliged to take technical and organizational precautions resulting in relatively high additional costs. The observed alkalinization of construction waste water affects open waters in developing countries, but also causes high cost for western companies, urged to install expensive setups for durable remediation. We developed a cheap and easy-to-maintain proof-of-concept micromotor based solution for pH remediation of alkaline concrete wash water. Within this process we use for the first time spray coating to achieve asymmetrization in micromotor fabrication. For this, we fabricate citric acid coated CaCO_3_ and MnCO_3_ micromotors and characterize them carefully. We studied their motion in water, in sodium hydroxide solution and in diluted concrete wash water (CWW). The effect these motors have on the neutralization of CWW was observed in bulk and on the micro scale, visualizing the obtained pH changes through anthocyanin indicator switches occurring in the vicinity of the micromotors. The reaction occurring between CaCO_3_ and citric acid helps in producing a buffering effect which in turn contributes to a controlled dosage of citric acid for neutralization. Since this reaction happens on a rather small scale, mostly niche applications are envisioned. For application of this POC strategy at large construction sites a smart technical implementation is required, however, the principle of combining acid and carbonate strategies is easy to handle and therefore highly promising. 

## Supplementary Materials

The following are available online at https://www.mdpi.com/2079-4991/10/7/1408/s1. The following are available in supplementary information. Video S1: Motion of the CA@CaCO_3_ and CA@MnCO_3_ micromotors in DI water. Video S2: Motion of the CA@CaCO_3_ and CA@MnCO_3_ micromotors in CWW of pH 9. Video S3: Motion of the CA@CaCO_3_ and CA@MnCO_3_ micromotors in NaOH solution of pH 9. Video S4: Microscopic neutralization of CWW by CA@CaCO_3_ and CA@MnCO_3_ micromotors in presence of anthocyanin dye. Figure S1: Pictorial scheme representing the contamination of groundwater due to infrastructural projects. Table S1: Cost overview of different methods used for neutralization. Figure S2: Detailed scheme for the synthesis of CaCO_3_ and MnCO_3_ microparticles. Figure S3: FTIR spectrum of as synthesized CaCO_3_ and MnCO_3_ particles. Figure S4: XRD patterns of as synthesized CaCO_3_ and MnCO_3_ particles with corresponding JCPDS data. Figure S5: Image of CaCO_3_ and MnCO_3_ particles in different pH solutions. Zeta potential and size of the CaCO_3_ and MnCO_3_ particles at different pH. Table S2: Conductivity values of MQ water, NaOH (pH 9 and 11) and concrete wash water (CCW) (pH 9 and 11).

## Abbreviations

The following abbreviations are used in this manuscript:

CWW	Concrete Wash Water
XRD	X-ray Diffraction
SEM	Scanning Electron Microscope
FTIR	Fourier Transform Infrared spectroscopy
CA	Citric acid
JCPDS	Joint Committee on Powder Diffraction Standards
POC	Proof of concept
