# Supplementary material for: Carbonate Micromotors for Treatment of Construction Effluents
Source: Nanomaterials (Basel). 2020 Jul 19;10(7):1408. doi: 10.3390/nano10071408 (PMC7407598; doi:10.3390/nano10071408)
Supplement: Supplementary file 1 [file nanomaterials-10-01408-s001.zip › Carbonate_motors_to_remediate_raised_pH_values_in_building_effluents (2).pdf]

Supplementary Information
Carbonate micromotors for treatment of construction effluents

Purnesh Chattopadhyay,[1] Priyanka Sharan,[1] Andrej Berndt[2] and Juliane Simmchen[1]
[1]Chair of Physical Chemistry, TU Dresden, 01062 Dresden, Germany
[2]Implenia, Zurich, Switzerland

May 21, 2020

List of Videos:
Video S1 Motion of the CA@$CaCO_3$ and CA@$MnCO_3$ micromotors in DI water.
Video S2 Motion of the CA@$CaCO_3$ and CA@$MnCO_3$ micromotors in CWW of pH 9.
Video S3 Motion of the CA@$CaCO_3$ and CA@$MnCO_3$ micromotors in NaOH solution of pH 9.
Video S3 Microscopic neutralization of CWW by CA@$CaCO_3$ and CA@$MnCO_3$ micromotors in presence of anthocyanin dye.

S1 Problem Statement - Controlled Contamination of ground water due to tunnel excavation

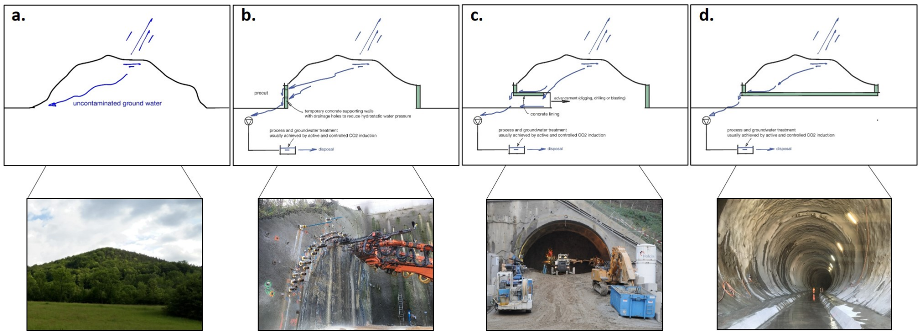

Figure S1: a. A beautiful mountain from Jena, Germany. Infrastructure projects such as digging a tunnel through mountains has an impact on hydrological subsystems of environment and can contaminate the ground water. b. For a tunnel project, first, temporary support walls made of concrete with drainage holes is used to reduce the hydrostatic pressure. Water coming in contact with the concrete becomes alkaline and hence the ground water needs adequate treatment. c. A finished precut with advancing tunnel excavation. d. Finished excavation with first shell of concrete lining applied as temporary support. The ground water passes over the sprayed concrete surfaces and most of time the pH reduction is obtained by active and controlled CO_2 induction.

Method	Material Cost to neutralize 1000 L of CWW (in euro)	Advantage	Disadvantage
†Dilution	-	-	Amount of water required is very high. 1 L of CWW (pH 12) requires 100000 L of tap water to neutralize.
†CO_2 pump	0.35*	High level of control in neutralization	External power supply is required.
†Strong Acids (HCl, HNO_3, H_2SO_4 etc.)	>2.80	External power supply is not required. There is no initial set up cost.	Careful handling required. Very hard to control the pH.
†Weak Acids (Citric acid, Oxalic acid etc.)	>2.80	External power supply is not required. There is no initial set up cost.	Very hard to control the pH.
‡Citric acid and MCO_3 hybrid (Present study)	>3.92 [M=$CaCO_3$] >3.78 [M=$MnCO_3$]	Easy to handle and to control the pH. No external power supply is required.	Needs to be tested at the industrial scale.

* Setup cost is not considered. †Taken from reference.[5] ‡Cost calculation is based on macroscopic neutralization experiments (cost evaluated for 12 mL CWW was correlated for 1000 L CWW)(Figure 6c). Industrial prices of citric acid (0.69 Euro/Kg) and carbonates ($CaCO_3$: 0.23 Euro/Kg, $MnCO_3$: 0.94 Euro/Kg) were considered for the cost evaluation.

Table S1: Overview of different methods used for neutralization of CWW

S2 Synthesis of $CaCO_3$ and $MnCO_3$

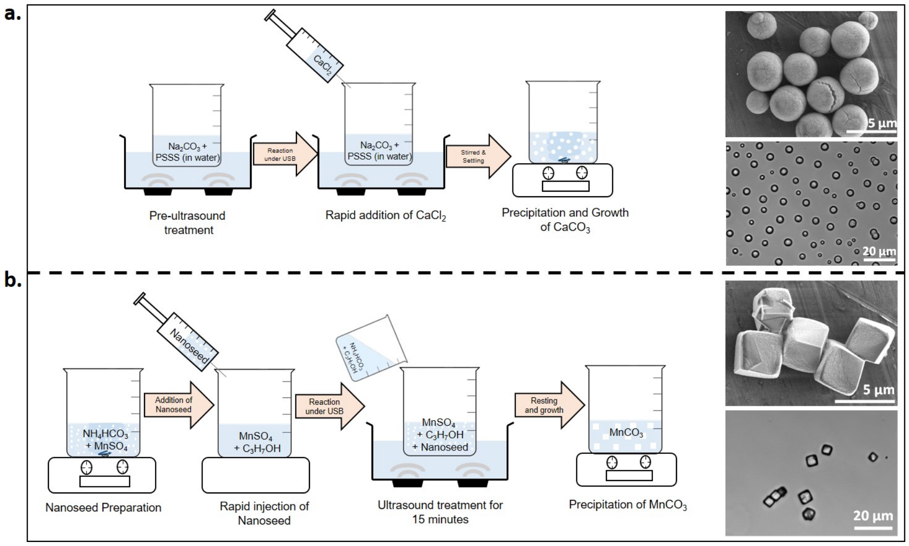

Figure S2: Detailed scheme for the synthesis of a.$CaCO_3$ b.$MnCO_3$.

Figure S2a and S2b shows a scheme of synthesis of $CaCO_3$ spheres and $MnCO_3$ cubes. In the right-hand side of the figure the corresponding SEM and optical images are shown which confirms the formation of the spherical and cubic morphology of the synthesized particles.

S3 Characterization of Carbonate particles

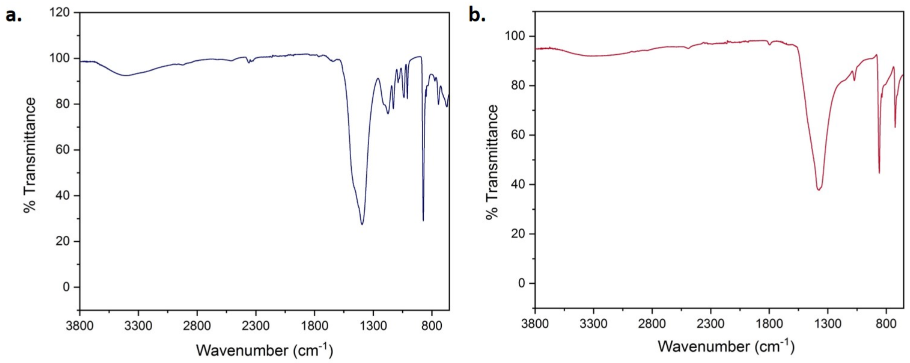

Figure S3: FTIR spectrum of as synthesized particles a. $CaCO_3$ b. $MnCO_3$

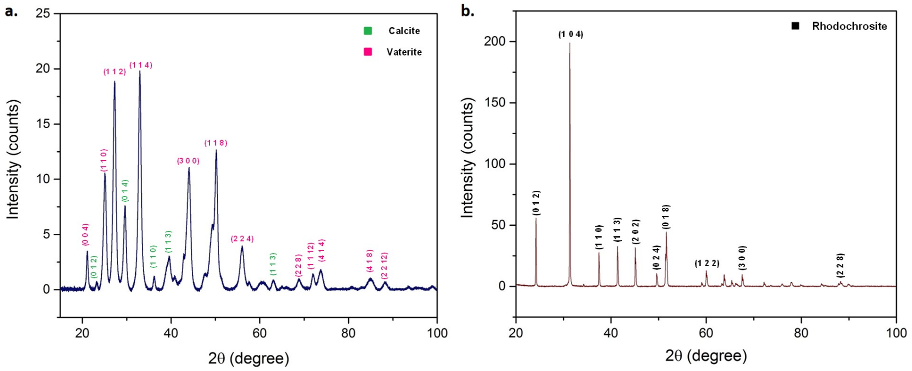

Figure S4: XRD patterns of as synthesized a. $CaCO_3$ b. $MnCO_3$ particles with corresponding JCPDS data.

The FTIR spectrum of the carbonate particles can be seen from Figure S3. $CaCO_3$ shows a characteristic absorption peak at 873 and 674 cm^{-1}. A small peak at 1088 cm^{-1} and a broad peak at 1400 cm^{-1} corresponds to the absorption peaks of symmetric and asymmetric stretching of carbonate groups (see Figure S3a).[2] For $MnCO_3$ (Figure S3b), sharp peaks at 725 and 860 cm^{-1} and a broad band at 1400 cm^{-1} corresponds to the characteristic vibrational bands of carbonate group.[4,7]

X-ray diffraction of the prepared samples was done to determine the crystalline phase of the prepared materials. $CaCO_3$ exists in three crystalline states Calcite,

aragonite, and vaterite.[3] The as-synthesized $CaCO_3$ showed mixed phases of calcite (marked in green with corresponding JCPDS file (C5-586) in Figure S4a) and vaterite (marked in pink with corresponding JCPDS file (C33-268) in Figure S4a). The $MnCO_3$ existed in Rhodochrosite form as the XRD pattern showed good agreement with its JCPDS file (C7-268) shown in Figure S4b.

S4 Measurement of size and zeta potential of the micromotors at different pH

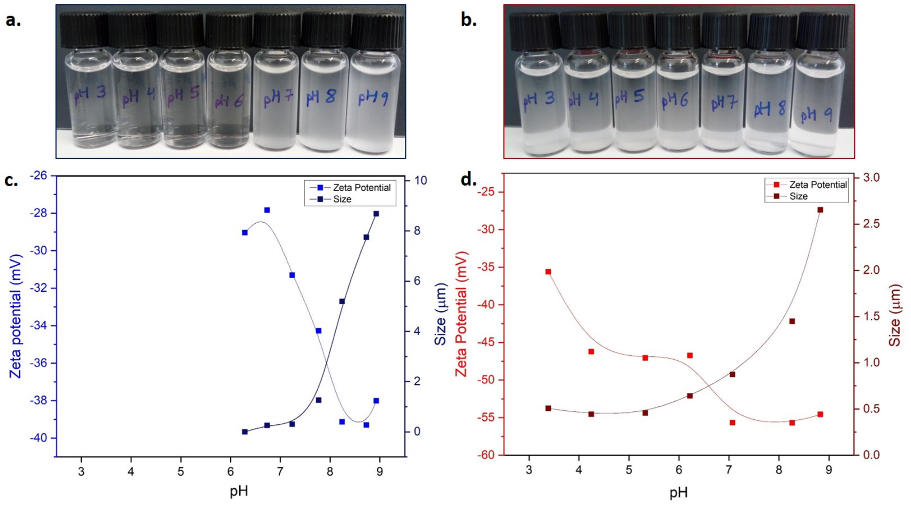

Figure S5: Image of a. $CaCO_3$, b. $MnCO_3$ particles in different pH solutions. zeta potential and size of the c. $CaCO_3$ d. $MnCO_3$ particles at different pH.

The size and zeta potential of the micromotors was measured in auto-titration mode using Zetasizer Nano ZSP (as seen in Figure S5). A mixture of carbonate particles and NaOH solution (pH 9) was titrated with 0.1 M HCl from pH 9 to pH 3. Size and zeta potential was measured automatically by the DLS instrument attached to the autotitrator at every 0.5 pH unit interval. It can be observed from both Figure S5c and S5d that the zeta potential value increases while the size of the particles decreases with the reduction of pH.
As the pH approaches to 6, the particle size reduces to zero due to complete dissolution in case of $CaCO_3$, causing abortion of any measurements after that point. This can also be reflected with the images shown in Figure S5a, here same amount of particles were added in an equal volume of buffer solution of correlating pH (pH 3-9). The vials were sonicated and imaged. As can be seen the vials with solutions below pH 6 are transparent showing complete dissolution of the $CaCO_3$ particles.

From the measurements of $MnCO_3$, despite the trend is similar to $CaCO_3$, the size of the particles never reduces to zero in this pH range. The visual bulk images (Figure S5b) also confirms that the particles do not dissolve even at acidic pH.

S5 Measurement of conductivity of different mediums used for motion studies

Medium	Conductivity (mS/cm)
MQ Water	0.023
NaOH(pH 9)	3.15
CCW(pH 9)	0.189
NaOH(pH 11)	8.83
CCW(pH 11)	6.283

Table S2: Conductivity values of MQ water, NaOH (pH 9 and 11) and concrete wash water (CCW) (pH 9 and 11)

The conductivity measurements of the mediums used for motion studies were done using Zetasizer (Table S2). 100 nm silica particles were used as tracers for these experiments. It can be seen from the table that the conductivity for MQ water is very low. For both NaOH and CWW (pH 9) the conductivity is high which corresponds to high ionic strength and this explains the slightly lower speed of motors (Figure 4b).[1,6] If the pH is further increased to 11 for NaOH and CWW, the conductivity increases drastically, thus limiting the motion of the motors.

References

[1] Aidan Brown and Wilson Poon. Ionic effects in self-propelled Pt-coated Janus swimmers. *Soft Matter*, 10(22):4016–4027, 2014.

[2] Guo Bin Cai, Shao Feng Chen, Lei Liu, Jun Jiang, Hong Bin Yao, An Wu Xu, and Shu Hong Yu. 1,3-diamino-2-hydroxypropane-N,N,N',N'-tetraacetic acid stabilized amorphous calcium carbonate: Nucleation, transformation and crystal growth. *CrystEngComm*, 12(1):234–241, 2010.

[3] Helmut Cölfen. Precipitation of carbonates: recent progress in controlled production of complex shapes. *Current Opinion in Colloid & Interface Science*, 8(1):23 – 31, 2003.

[4] Hack Keun Lee, Daisuke Sakemi, Roman Selyanchyn, Cheal Gyu Lee, and Seung Woo Lee. Titania nanocoating on $MnCO_3$ microspheres via liquid-phase deposition for fabrication of template-assisted core-shell- and hollow-

structured composites. *ACS Applied Materials and Interfaces*, 6(1):57–64, 2014.

[5] Siltbuster ltd. Solutions for the Concrete Industry. (4737424).

[6] Walter F. Paxton, Paul T. Baker, Timothy R. Kline, Yang Wang, Thomas E. Mallouk, and Ayusman Sen. Catalytically induced electrokinetics for motors and micropumps. *Journal of the American Chemical Society*, 128(46):14881–14888, 2006.

[7] Lei Wang, Yuanwei Sun, Suyuan Zeng, Chuansheng Cui, Haibo Li, Shuling Xu, and Huaisheng Wang. Study on the morphology-controlled synthesis of $MnCO_3$ materials and their enhanced electrochemical performance for lithium ion batteries. *CrystEngComm*, 18(41):8072–8079, 2016.
